# Unexpected high seroprevalence of hepatitis E virus in patients with alcohol-related cirrhosis

**DOI:** 10.1371/journal.pone.0224404

**Published:** 2019-10-24

**Authors:** Anabella C. Fantilli, Julieta Trinks, Sebastián Marciano, Fabián Zárate, Domingo C. Balderramo, Maribel G. Martínez Wassaf, Leila Haddad, Adrián Gadano, José D. Debes, María B. Pisano, Viviana E. Ré

**Affiliations:** 1 Instituto de Virología “Dr. J. M. Vanella”, Facultad de Ciencias Médicas, Universidad Nacional de Córdoba, Córdoba, Argentina; 2 Consejo Nacional de Investigaciones Científicas y Técnicas (CONICET), Buenos Aires, Argentina; 3 Instituto de Medicina Traslacional e Ingeniería Biomédica (IMTIB)—CONICET—Instituto Universitario del Hospital Italiano (IUHI)—Hospital Italiano (HIBA), Buenos Aires, Argentina; 4 Sección de Hepatología, Hospital Italiano de Buenos Aires, Buenos Aires, Argentina; 5 Departamento de Investigación, Hospital Italiano de Buenos Aires, Buenos Aires, Argentina; 6 Hospital Córdoba, Córdoba, Argentina; 7 Departamento de Gastroenterología, Hospital Privado Universitario de Córdoba, Instituto Universitario de Ciencias Biomédicas de Córdoba, Córdoba, Argentina; 8 LACE Laboratorios, Córdoba, Argentina; 9 Department of Medicine, Division of Infectious Diseases and International Medicine, University of Minnesota, Minneapolis, Minnesota, United States of America; Medizinische Fakultat der RWTH Aachen, GERMANY

## Abstract

**Introduction:**

Little is known about hepatitis E virus (HEV) infection in patients with cirrhosis. The aim of the present study was to describe the frequency of HEV infection and associated risk factors in patients with cirrhosis from Argentina.

**Materials and methods:**

We evaluated HEV seroprevalence (IgG anti-HEV) and acute infections (IgM and RNA) in patients with cirrhosis (n = 140) vs. healthy controls (n = 300). Additionally, we compared the same outcomes in individuals with alcohol-related cirrhosis (n = 43) vs. patients with alcohol use disorder (without cirrhosis, n = 72).

**Results:**

The overall HEV seroprevalence in the cohort of subjects with cirrhosis was 25% (35/140), compared to 4% in the healthy control group [12/300; OR = 8; (95% CI = 4–15.99); p<0.05]. HEV seropositivity was significantly higher in alcohol-related cirrhosis compared to other causes of cirrhosis [39.5% vs. 12.4%; OR = 4.71; (95% CI = 1.9–11.6); p<0.05] and to healthy controls [OR = 15.7; (95% CI = 6.8–36.4); p = 0.0001]. The HEV seroprevalence in alcoholic-related cirrhosis vs. with alcohol use disorder was 39.5% vs. 12.5% [OR = 4.58; (95% CI = 1.81–11.58); p<0.001].

**Conclusion:**

We found a high seroprevalence of HEV in patients with cirrhosis and in individuals with alcohol use disorder. The simultaneous presence of both factors (cirrhosis + alcohol) showed more association to HEV infection. Larger studies with prospective follow up are needed to further clarify this interaction.

## Introduction

Hepatitis E virus (HEV) (specie *Orthohepevirus A*, genus *Ortohepevirus*, family *Hepeviridae*) is a non-enveloped virus with a positive sense single stranded RNA genome. It is one of the leading causes of acute viral hepatitis of enteric transmission worldwide, resulting in a spectrum of clinical manifestations from self-limiting to fulminant [1,2]. Eight genotypes have been described, from which 5 can infect humans (1–4 and 7); with 3 of them (3, 4 and 7) considered zoonotic [2,3].

Multiple studies from a variety of regions around the world, have reported that individuals with immunosuppression and/or chronic liver disease have higher HEV seroprevalence rates and a higher risk to develop chronic hepatitis compared to the general population [4–8]. Chronic HEV cases have mostly been observed in these populations when the infecting genotype is 3 (HEV-3) [9,10].

An association between HEV infection and progression to cirrhosis in immunocompetent individuals has also been described [11–14]. However, the underlying mechanisms of this interrelation are not yet clear. Up to our knowledge, there are only 4 previous studies that reported high HEV seroprevalence rates in patients with cirrhosis worldwide [15–18].

In South America, sporadic acute HEV cases have been informed and many studies have documented detection of HEV-3 RNA in a variety of sources, such as animal reservoirs (pigs and wild boars) and environmental matrices (surface waters and sewage) [3]. Its circulation has also been reported through serological surveys in many populations (general population, blood donors, HIV+ individuals, pediatric populations, dialyzed patients, solid organ transplant recipients, etc.) [3]. However, in this region and particularly in Argentina, no study has yet addressed the association of HEV infection to certain hepatic pathologies, such as cirrhosis.

In this context, the aim of the present study was to estimate the HEV seroprevalence in patients with cirrhosis and to evaluate associated risk factors.

## Materials and methods

### Study design

We performed a cross-sectional study during the years 2017 and 2018 in 1 medical centre from the city of Buenos Aires (private centre with attending patients on middle and high incomes) and 2 medical centres from Córdoba (1 private centre, with attending patients on middle and high incomes, and 1 public centre, with attending individuals on low incomes), all of them located in the central region of Argentina.

We evaluated HEV seroprevalence (presence of IgG anti-HEV) in patients with cirrhosis vs. healthy controls. Additionally, we compared values obtained in individuals with alcohol-related cirrhosis vs. patients with alcohol use disorder (AUD, without cirrhosis, as defined by [19]).

With the aim of detecting acute infections, we investigated the presence of IgM anti-HEV and RNA-HEV.

### Samples

a) Cases: patients with cirrhosis (n = 140): analysed for IgG and IgM anti-HEV, as well as HEV-RNA detection. The diagnosis of cirrhosis was based on liver biopsy or composite of clinical signs and findings provided by laboratory tests, endoscopy, abdominal Doppler ultrasound and radiologic imaging studies. Patients were categorized according to the cause of cirrhosis: viral hepatitis n = 23, alcoholic-related cirrhosis n = 43, primary biliary cholangitis n = 9, non-alcoholic steato-hepatitis (NASH) n = 24 and cirrhosis due to other causes n = 26 (autoimmune hepatitis, cryptogenic hepatitis, secondary biliary cirrhosis).

In 59 patients, biochemical parameters of liver inflammation and function (ALT, AST and bilirubin levels) were available.

b) Controls: healthy control group of the general population (n = 300): individuals who attended health-care centres from Córdoba city for a routine control, which were selected from the serum bank of the Instituto de Virología “Dr. J. M. Vanella”, and retrospectively analysed as a control group, for IgG anti-HEV detection.

c) Patients with AUD without cirrhosis (n = 72): These patients had fatty liver or liver without alterations (determined by abdominal Doppler ultrasound) and were analysed for IgG and IgM anti-HEV.

### Serological tests

IgG and IgM anti-HEV antibodies were tested by third generation ELISA assays, using commercial kits (Diapro, Italy), following the manufacturer’s instructions and as described before [3].

In brief, ELISA microplates were coated with HEV-specific, synthetic, conserved and immunodominant antigens, derived from the open reading frames 2 and 3 (ORF-2 and ORF-3) of HEV genome of genotypes 1–4. Test results were used to calculate the ratio of the sample (S) and the cut-off (CO) (S/CO). Samples with S/CO ratio bellow 0.9 were considered negative, between 0.9–1.1 as equivocal results, and above 1.1 as positive results.

### Molecular analyses

IgG and/or IgM anti-HEV positive samples were subjected to HEV molecular detection. For viral nucleic acids extraction, the QIAamp Viral RNA Kit (Qiagen GmbH, Germany) was used, following manufacturer’s instructions. To obtain cDNA, retrotranscription (RT) was carried out using *random hexamer primers* and the enzyme Reverse Transcriptase (ImPromII -Reverse Transcriptase- Promega). Genomic detection of HEV was performed with a nested-PCR protocol, amplifying a 348 bp fragment of the ORF-2 region for HEV 1–4 genotypes, previously described by [20], using the enzyme GoTaq (Promega) [20]. PCR products were analyzed by gel electrophoresis using TBE buffer and a 2% agarose gel containing GelRed (Biotium, Inc), following the manufacturer’s instructions, and visualized under UV light. The lower limit of detection for this PCR was 31.6 PID (pig infectious dose).

### Statistical analyses

Categorical variables are expressed as numbers and percentages. Continuous variables are expressed as median and range. To assess the association between individual variables and IgG anti-HEV seropositivity we used an independent t or χ2 test. The strength of association was estimated by means of Odds ratios (OR) and 95% confidence intervals (CIs). Statistical significance was defined at p<0.05. The statistical package Stata 13.0 was used.

The socioeconomic level (low-income and middle/high-income populations) was stratified following a classification based on the economic, social and educational level of each person, according to [21].

### Ethics statement

This study was approved by the Ethics Committee of the Health Ministry of the Province of Córdoba (CIEIS Hospital Privado Universitario de Córdoba, protocol Nº HP-4-231) and the Ethics Committee of the Hospital Italiano from Buenos Aires (protocol Nº E/127), Argentina. A written informed consent was obtained for each individual enrolled.

## Results

A total of 512 individuals were evaluated for the presence of HEV. The male-to-female ratio for patients with cirrhosis (n = 140) was 1.8/1, with a mean age of 61 years (23–88 years). The male-to-female ratio for healthy individuals (n = 300) was 0.3/1, with a median age of 35 years (20–78 years). The male-to-female ratio and the median age for patients with AUD (n = 72) was 8/1 and 51 years (27–67 years), respectively.

**Fig 1:**
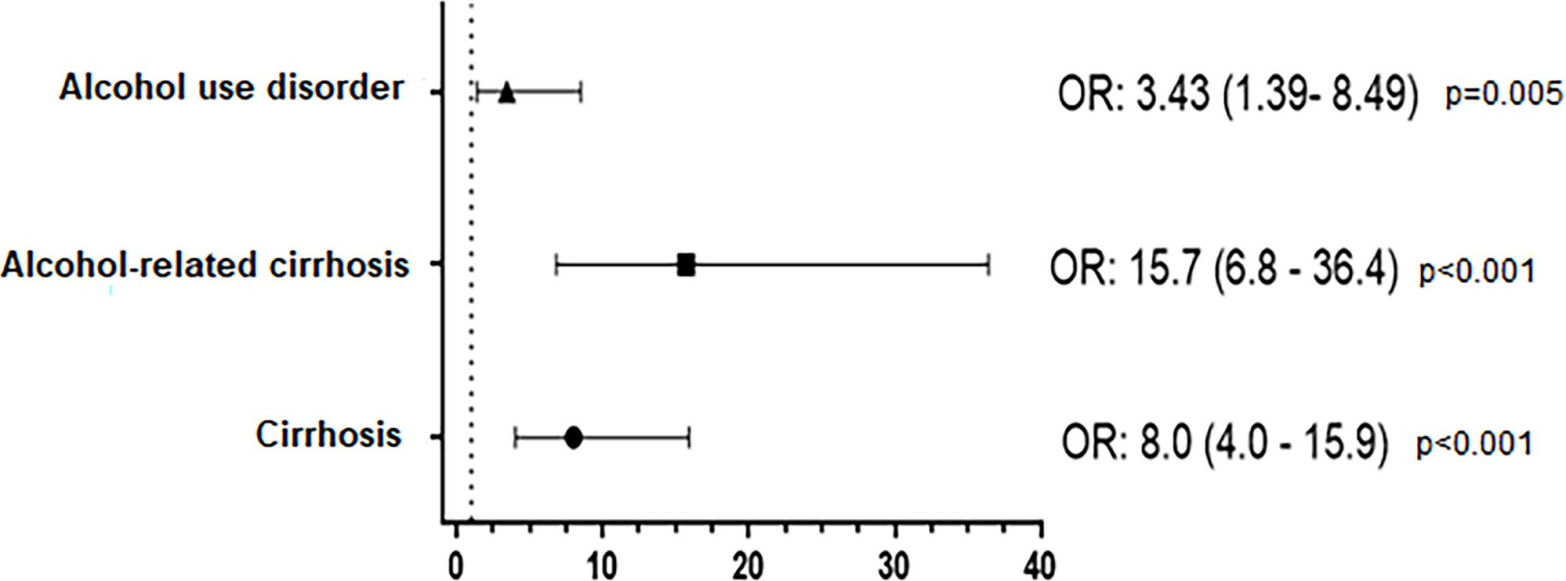
Associated risk factors with HEV seropositivity.

The IgG anti-HEV seroprevalence in patients with cirrhosis was significantly higher than in healthy controls (25% vs. 4%, OR = 8, 95% CI: 4–15.99, p<0.001) (Fig 1). Since the median age of both groups were different, and taking into account that seroprevalence could increase with age, we further analysed a subgroup of 93 healthy controls with a mean age of 55 years (46–78 years). The HEV seroprevalence obtained in this subgroup was 7.5%, maintaining the significant difference with the group of patients with cirrhosis. Added to this, as the male-to-female ratio was also quite different in patients and general population, we randomly selected a subgroup of 118 healthy controls with the same male-to-female rate (1.8/1), finding no significant difference between both groups (25% patients with cirrhosis vs. 4.2% healthy controls).

When stratifying samples according to geographical origin, HEV seroprevalence in individuals with cirrhosis were 18.5% in Cordoba and 33.9% in Buenos Aires, both significantly higher than the rates reported for general populations in those areas [4% vs. 18.5%; p<0.001 for Córdoba, and 9.5% (previously reported by [22]) vs. 33.9%; p<0.001 for Buenos Aires].

No significant differences were found in the HEV seroprevalences when analysing the samples according to the socioeconomic status.

Of the 35 IgG anti-HEV positive samples in patients with cirrhosis, 16 were reactive for IgM anti-HEV (45.7%). Three of the 140 samples (2.1%) resulted positive for HEV-RNA amplification, although the genotype could not be determined. There were no associations between biochemical parameters and the HEV serological and molecular status.

The seroprevalences of IgG anti-HEV according to the causes of cirrhosis are shown in Table 1. Overall, HEV seropositivity was significantly higher in alcoholic-related cirrhosis when compared to the general population (healthy control group) [OR = 15.7; (95% CI = 6.8–36.4); p <0.001 (Fig 1)] and to other causes of cirrhosis [39.5% vs. 12.4%; OR = 4.71; (95% CI = 1.9–11.6); p <0.05] (Table 1).

**Table 1:** IgG anti-HEV serostatus in patients with cirrhosis according to the causes of cirrhosis.

	Positive		Negative		Total^a^	
	n	%	n	%	n	%
Alcoholic-related cirrhosis^b^	17	39.5%	26	60.5%	43	34.7%
Viral hepatitis	3	13.0%	20	87.0%	23	18.6%
NASH	2	8.3%	22	91.7%	24	19.4%
Primariy biliary cholangitis	2	22.2%	7	77.8%	9	7.3%
Other causes ^c^	3	11.5%	23	88.5%	26	21.0%

NASH: non-alcoholic steato-hepatitis.

^a^Available data for 125 patients.

^b^p-value<0.05; OR = 4.71 (95% IC = 1.9–11.6), when comparing with all the other causes of cirrhosis.

^c^Autoimmune, cryptogenic and secondary biliary cirrhosis.

Due to this unexpected association between alcoholic-related cirrhosis and HEV, we later attempted to determine if alcohol consumption could itself be considered a risk factor for HEV infection. Nine samples out of 72 AUD without cirrhosis (12.5%) yielded a positive result for anti-HEV IgG. This percentage was statistically higher than the obtained in the healthy control group, suggesting a positive association between alcohol consumption and HEV seropositivity [12.5% vs. 4%; OR = 3.43; (95% CI = 1.39–8.49, p = 0.005) (Fig 1)]. IgM anti-HEV was detected in only 1 out of the 9 IgG anti-HEV (+) samples analysed (11%).

RNA-HEV could not be tested in these samples likely due to storing conditions being sub-optimal to carry out advanced molecular detection.

When comparing the HEV seroprevalence in individuals with alcoholic-related cirrhosis and AUD we found a statistically significant difference (39.5% vs. 12.5%; p = 0.0008), being more likely to be HEV seropositive in patients with alcoholic-related cirrhosis [OR = 4.58; (95% CI = 1.81–11.58)].

## Discussion

An association between HEV infection and liver cirrhosis has been reported in several studies; yet there is a lack of clarity of related risk factors and prognostic significance. It has been reported that HEV infection could negatively impact the prognosis of patients with chronic liver diseases [23,24]. However, in Latin America there is only one recent report from Brazil about HEV infection in individuals with chronic liver disease [17], and no data documented in Argentina.

In our study, we found that seropositivity for IgG anti-HEV in individuals with cirrhosis was 7 times higher than that of healthy controls. It is known that HEV seroprevalence varies according to the geographical region (even within a country) and the age of the individuals analyzed. This is the case of Argentina, where slightly variations in seroprevalence values of general population have been documented among different studied regions [3] and different age groups (higher seroprevalences in individuals older than 46 years) [25]. Our samples originated from two different regions within Argentina. When samples were analyzed stratified according to their region of origin, we still found significant differences among individuals with cirrhosis and those of general population in each region. The same was observed when we compared individuals with cirrhosis and a subgroup of healthy controls with similar ages and with the same male-to-female ratio, indicating that, in this setting, the high anti-HEV IgG positivity rate is associated with parameters other than age or sex, probably associated with the presence of cirrhosis.

Previous studies have reported higher HEV seroprevalence rates in patients with cirrhosis, such as in Spain (17.5%), China (6.5%), Turkey (25.7%) and recently in Brazil (13.2%), when compared to those obtained in healthy individuals and patients with chronic hepatitis (without cirrhosis) from the four regions [15–18]. Interestingly, a study from Spain in HIV infected patients, found that liver cirrhosis was the only risk factor related to the presence of antibodies against HEV [26]. In Nepal, a recent research has also documented an association of HEV seropositivity with an advanced stage of liver fibrosis [27].

It is not totally clear why cirrhosis would predispose to HEV seropositivity. Several studies have reported that cirrhosis-associated immune dysfunction can lead to alterations in innate and acquired immunity, both at an intrahepatic and systemic level. This could, in theory, explain why patients with cirrhosis may be prone to contract HEV infection [28,29]. On the other hand, the significantly higher seroprevalence of IgG anti-HEV in patients with cirrhosis compared to the seroprevalence of those with chronic hepatitis (without cirrhosis) found in the studies from Spain and China [15,16], could be an indication that HEV infection contributes to the progression of chronic liver disease to cirrhosis.

Interestingly, we found an unexpected high seroprevalence of HEV specifically in patients with alcoholic-related cirrhosis. This group of patients expressed an increased risk of HEV infection, 14 times higher than the control group, and 3 times higher than individuals with other causes of cirrhosis (and with similar ages). To our knowledge, only one previous study from China has reported a significant association between the seroprevalence of HEV infection and alcoholic-related cirrhosis, in which a correlation between HEV infection, hepatitis B and autoimmune hepatitis was also found [16].

Previous studies have proposed alcohol consumption to be considered an important risk factor for HEV, as it contributes to the clinical expression of the infection and the severity of hepatitis [30]. Moreover, a study that reported a HEV-3 outbreak on a cruise ship, identified alcohol consumption as a key risk factor, suggesting that excess alcohol consumption could compromise hepatic function and predispose to symptomatic hepatitis E infection [31]. Interestingly, a recent report shows that binge alcohol consumption can induce bacterial translocation into the blood [32]. As transmission of HEV is thought to be fecal-oral via the gut system, it is not unreasonable to speculate that upon alcohol drinking there could also be a component of viral, in addition to bacterial, translocation allowing for the exposure to HEV.

In addition, it is known that excessive alcohol consumption can induce subclinical liver disease (steatosis and/or fibrosis), which increases the susceptibility of the organ to develop viral infections [30,31,33]. This could lead to more frequent HEV infections (and a consequent increased seroprevalence), as well as a greater likelihood of symptomatic infections (as reported by [30]).

Our study found a high seroprevalence of IgM anti-HEV in individuals with alcoholic-related cirrhosis, suggesting recent infections in the studied population, although there were not clinical signs of acute infection and biochemical parameters were within reference values. Therefore, these results should be interpreted with caution.

Our study has several limitations, including: a) the impossibility of genotyping the RNA amplified, which was probably due to a low viral load, b) the relatively low number of samples analyzed, together with the low number of variables analyzed, which did not allow multivariate analyses discarding possible confounders that could explain our findings more accurately, c) the lack of sequential testing which would allow to determine whether alcoholic-related cirrhosis is a predisposing factor for HEV infection or if HEV infection contributes to progression of liver fibrosis to cirrhosis.

Larger studies focused on a higher number of individuals with alcoholic-related cirrhosis with prospective follow up are needed to further clarify this interaction. Moreover, evaluation of the cellular systemic immune response and liver local immune response with sequential monitoring of HEV-RNA in individuals with cirrhosis and liver-related cirrhosis would help to clarify the association and identify potential prevention points.
